# The Activation of the NF-κB Pathway in Human Adipose-Derived Stem Cells Alters the Deposition of Epigenetic Marks on H3K27 and Is Modulated by Fish Oil

**DOI:** 10.3390/life14121653

**Published:** 2024-12-12

**Authors:** Jussara de Jesus Simao, Andressa França de Sousa Bispo, Victor Tadeu Gonçalves Plata, Ana Beatriz Marques Abel, Raphael Justa Saran, Júlia Fernandes Barcella, João Carlos Cardoso Alonso, André Valente Santana, Lucia Maria Armelin-Correa, Maria Isabel Cardoso Alonso-Vale

**Affiliations:** 1 Post-Graduate Program in Chemical Biology, Institute of Environmental Sciences, Chemical and Pharmaceutical, Federal University of São Paulo—UNIFESP, Diadema 09913-030, Brazil; jussara.simao@unifesp.br (J.d.J.S.); andressa.franca@unifesp.br (A.F.d.S.B.); victor.plata@unifesp.br (V.T.G.P.); lucia.correa@unifesp.br (L.M.A.-C.); 2 Post-Graduate Program in Nutrition, Paulista School of Medicine, Federal University of São Paulo—UNIFESP, Sao Paulo 04023-062, Brazil; anabeatrizmarquesabel@gmail.com; 3 Department of Biological Sciences, Institute of Environmental Sciences, Chemical and Pharmaceutical, Federal University of São Paulo—UNIFESP, Diadema 09913-030, Brazil; raphael.justa@unifesp.br (R.J.S.); julia.barcella@unifesp.br (J.F.B.); 4 Paulínia Municipal Hospital, Paulínia 13140-000, Brazil; jccalonso@hotmail.com; 5 Post-Graduate Program in Interdisciplinary Surgical Science, Paulista School of Medicine, Federal University of São Paulo—UNIFESP, Sao Paulo 04023-062, Brazil; andrevsantana@yahoo.com.br

**Keywords:** adipose tissue, mesenchymal stem cells, lipopolysaccharide, inflammation, H3K27ac, H3K27me3, KDM6B, n-3 PUFA

## Abstract

Background: Chronic low-grade inflammation in obesity is linked to white adipose tissue (WAT) dysfunction. Plasma lipopolysaccharide (LPS) activates Toll-like receptor 4 (TLR4), triggering NF-κB and worsening these disturbances. Previously, we showed that histone H3 lysine 27 (H3K27) epigenetic modifications affect WAT gene expression in high-fat-diet mice, identifying key pathways in adipose-derived stem cells (ASCs). This study explores whether NF-κB influences H3K27 modifiers in human ASCs and evaluates fish oil (FO) as a modulator. Methods: Human visceral WAT ASCs were stimulated with LPS and treated with FO enriched with eicosapentaenoic acid (EPA). Flow cytometry, PCR array, RT-PCR, and Western blot assays were used. Results: LPS increased NF-κB activity, elevating KDM6B demethylase levels and H3K27 acetylation. These epigenetic modifications in LPS-stimulated ASCs were associated with persistent changes in the expression of genes involved in adipogenesis, metabolic regulation, and inflammation, even after LPS removal and cell differentiation. FO mitigated these effects, reducing H3K27 acetylation and promoting methylation. Conclusions: FO demonstrates potential in modulating inflammation-induced epigenetic changes and preserving adipocyte function.

## 1. Introduction

White adipose tissue (WAT) displays high plasticity in response to the individual’s diverse metabolic demands. While mature adipocytes primarily drive this plasticity, adipose-derived stem cells (ASCs), found within the vascular stromal fraction of WAT, facilitate WAT expansion by differentiating into new adipocytes. This process is fundamental to allow the healthy adjustment of lipid reserves, thereby minimizing any disruptions to WAT homeostasis [1,2].

During adipogenesis, epigenetic alterations involving histone 3 lysine 27 (H3K27) are critical for silencing the genes responsible for the commitment of ASCs to other cellular fates, such as chondrogenesis and osteogenesis, as well as for the activation of the transcription factors critical for adipogenesis [3,4,5]. The Enhancer of Zeste Homolog 2 (Ezh2) is responsible for the trimethylation of H3K27 (H3K27me3), resulting in gene silencing, though its effects on gene expression can vary, depending on the context [4,6,7]. These effects can be reversed by a couple of histone lysine demethylases, KDM6A/UTX and KDM6B/JMJD3, which were found to inhibit adipogenesis and promote the osteogenic differentiation of human mesenchymal stem cells (hMSCs) [8,9]. On the other hand, histone acetylation, such as H3K27ac, leads to transcriptional activation and is promoted by acetyltransferases CREBBP and EP300 (CBP/p300). CBP/p300, in the PPARγ complex, is the main enzyme that activates gene transcription. It can also increase the expression of CEBPα and PPARγ to promote adipogenesis, as recently reviewed [10].

ChIP-seq assays have shown that the entire NF-kB transcription factor family binds to the EZH2 promoter in human lymphoblastoid cell lines and in melanoma and retinoblastoma cancer [11,12,13,14]. The encyclopedia of DNA Elements (ENCODE) project revealed multiple NF-kB (RelA) binding sites not only in the EZH2 promoter region but also in the promoters of other H3K27 modifiers like Crebbp, Ep300, Kdm6a, and Kdm6b. In ASCs, the transcriptional regulation of histone H3 modifiers by NF-κB is still largely unknown. In obese individuals, elevated levels of circulating lipopolysaccharide (LPS) significantly activate NF-κB in WAT [15,16]. We hypothesize that in a low-grade inflammatory environment, typical of obesity, NF-κB modulates the expression of histone modifiers in these progenitor cells.

Consistent with this hypothesis, we recently demonstrated a strong correlation between H3K27 epigenetic modifications and high-fat diet (HFD)-induced obesity in both ASCs [17] and WAT [18] from mice. However, in the context of chronic inflammatory conditions associated with obesity, the specific impact of H3K27 marks on WAT, particularly on ASCs, as well as the role of NF-κB in this process in humans, remains poorly understood.

Extensive research has underscored the remarkable benefits of long-chain polyunsaturated fatty acids n-3 (n-3 PUFA), notably eicosapentaenoic acid (EPA) and docosahexaenoic acid (DHA), abundant in fish oil (FO), in mitigating obesity-related chronic inflammatory conditions [19,20,21,22,23]. Building upon our prior investigations, which illuminated the efficacy of EPA-rich FO supplementation at a 5:1 EPA:DHA ratio, we demonstrated, in mice with HFD-induced obesity that were administered with this formulation, marked reductions in body weight and adipose mass, alongside improved adipocyte function and marked improvements in obesity-related metabolic and endocrine dysfunctions [24,25,26,27]. Furthermore, our recent study showed that FO significantly attenuated the HFD-induced alterations in the WAT gene expression profile and exhibited potential in mitigating the epigenetic changes triggered by inflammation associated with obesity in mice [18].

In this study, we used an in vitro approach and visceral human WAT samples to investigate how the activation of the NF-κB pathway influences the deposition of epigenetic marks on H3K27 in ASCs, and whether this process is modulated by FO.

## 2. Materials and Methods

### 2.1. Human Subjects

The entire protocol and the use of samples were approved by the Research Ethics Committee of UNIFESP (CEP/UNIFESP Project No.: 0268/2022). Visceral WAT (abdominal region) was collected from patients undergoing gastric surgery at the Municipal Hospital of Paulínia-SP, after having signed the Informed Consent Form (ICF). Four male patients were between 30 and 50 years old and overweight (25 < BMI < 30).

The visceral WAT samples were processed in the Adipose Tissue Physiology and Epigenetics Laboratory of UNIFESP, following the protocol described [28]. After the dissection and fragmentation of the WAT, the samples were placed in a digestion buffer (Dulbecco’s modified Eagle’s medium—D’MEM/HEPES 20 mM/BSA 4%, collagenase II [Sigma Chemical, St. Louis, MO, USA]—1.0 mg/mL, pH 7.40) for tissue digestion by collagenase and cell isolation [29]; the incubation took place for approximately 45–60 min at 37 °C in a water bath with orbital shaking (130 rpm). The samples were then filtered through a fine-mesh plastic sieve (which retains tissue debris and undigested vessels) and the volume was completed to 25 mL of EHB buffer (EARLE/HEPES salts 25 mM, BSA 1%, sodium pyruvate 1 mM, without glucose, pH 7.45, 37 °C) in a 50 mL Falcon flask. The filtrate was centrifuged (400 G, 1 min) and then divided into two fractions: 1. the upper layer or supernatant, which contains the isolated mature adipocytes; 2. the stromal cell fraction (SVF) or remnant of the filtrate, which was subjected to a new centrifugation (1500 G for 10 min). The fraction was contained in the pellet (lower layer) after centrifugation. The material was then resuspended and washed twice with 25 mL of EHB buffer, following the same centrifugation steps as for the adipocytes and the SVF (which contains ASCs).

### 2.2. Isolation and Cell Culture

We adhered to a previously established protocol with modifications [30]. The cell pellet (SVF) obtained from the digestion of the visceral WAT sample was resuspended in culture medium [D’MEM Han’s F-12, supplemented with 10% fetal bovine serum (FBS) and 10 mL/L penicillin/streptomycin (Gibco, Grand Island, NY, USA) and plated in culture dishes, which were incubated in a 5% CO_2_ environment at 37 °C. The culture medium was refreshed every two days until the cells reached 70–80% confluence. Following this period, the medium was discarded and the plates were rinsed with PBS. The final step in isolating the ASCs involved selecting the adherent SVF population. The cells were trypsinized for the first time (passage 1, P1), resuspended in the same culture medium, and transferred to larger plates for further expansion, continuing until they reached 70–80% confluence again. Cell concentration was assessed using a Neubauer chamber, and the cells were replated (P2), consistently maintaining a maximum confluence of 80% during the passages. Between P2 and P5, the cells were prepared for experiments at a density of 1 × 10^5^ cells per well in 6-well plates (35 mm). When they reached 85–90% confluence (during proliferation) or after differentiation as indicated in the text, the cells were treated (or not) with LPS and/or FO and/or JSH-23 (4-Methyl-N1-(3-phenylpropyl) benzene-1,2-diamine) for 72 h.

### 2.3. Cell Treatments

The FO used in this study was rich in EPA (EPA/DHA ratio 540/100 mg/g, or 5:1, HiOmega-3, Naturalis, São Paulo, Brazil). FO was added to the cultures for 72 h, as indicated in the text, at a concentration of 50 μM and dissolved in ethanol (vehicle) not exceeding 0.05% in the controls. The cells were stimulated with 1 µg/mL of LPS (*E. coli* 05:88; Sigma), diluted in a culture medium. NF-κB inhibitor JSH-23 [4-Methyl-N1-(3-phenylpropyl) benzene-1,2-diamine, SigmaAldrich, St. Louis, MO, USA], a cell-permeable diamine compound that selectively blocks the nuclear translocation of the p65/p50 NF-κB dimer and its transcriptional activity without affecting the degradation of IκB-α, was added to the cultures diluted in DMSO (Dimethyl sulfoxide, Synth, São Paulo, Brazil). The dose used was based on previous studies [31].

### 2.4. Adipogenic Differentiation

For differentiation, the cells were cultured until reaching 100% confluence. At 100% confluence (day D0), the cells were stimulated to differentiate by treatment with an adipogenic cocktail composed of D’MEM/Han’s F12 plus 0.5 mM IBMX (3-isobutyl-1-methylxanthine), 0.1 μM dexamethasone, 0.5 μM human insulin, 2 nM T3, 30 μM indomethacin, 17 μM pantothenate, 33 μM biotin, 1 μM Rosiglitazone, 1 mg/mL Apo-transferrin, 2% FBS, and antibiotics (1% penicillin–streptomycin) for 4 days (D4) in the absence of FO or LPS or JSH23. The cells were subjected to the extraction of total RNA and proteins for the analysis of the expression of markers of histone modifying enzymes and the NF-κB pathway.

### 2.5. Immunophenotyping

Human ASC markers have been well characterized. We used the following markers: positive staining for CD73 and CD90 and negative staining for CD45 and CD31 [28]. We used BD Bioscience antibodies that were already labeled with fluorophore. For characterization by the immunophenotyping of ASCs, we incubated a concentration of 1 × 10^6^ cells resuspended in 100 μL, with the respective antibodies, for 30 min protected from light. The cells were fixed and resuspended in 500 ul of PBS with 3% BSA. The cells were evaluated in FACS Canto II equipment with the acquisition of at least 1 × 10^4^ events.

### 2.6. RNA Extraction and Gene Expression Analysis by Real-Time qPCR and PCR Array

The total RNA for the RT-PCR assays was extracted from the ASCs with a Trizol reagent (TRIzol, Invitrogen, Carlsbad, CA, USA), according to the supplier’s instructions, and for the PCR array assays, RNA was isolated using an RNA extraction kit and following the manufacturer’s instructions: RNeasy (QIAGEN, Hilden, Germany)^®^ Lipid Tissue Mini Kit (Cat. No. 74804). After both extractions, the quantity (ng/mL) and purity (260/280 and 260/230 ratios) of the total RNA was assessed with NanoDrop spectrophotometer (Thermo Scientific, Walthamm, MA, USA). The total RNA was reverse transcribed for complementary DNA (cDNA) synthesis, starting with 1 μg of total RNA and an oligo dT primer. The reaction was catalyzed by reverse transcriptase (SuperScript III, Invitrogen Life Technologies) in the presence of dNTPs. For the RT-qPCR experiments, gene expression was assessed using a Rotor-Gene (Qiagen) and SYBR Green as the fluorescent dye, following the manufacturer’s instructions, and the data analysis was performed using the 2^(-ΔΔCt) method [32]. The results are presented as the ratio of the target gene expression to the housekeeping gene (GAPDH). The following primers were used: *CREBBP* (*CBP*) (5′-3′ sense: GAAACCAACAAACCATCCTGG; 5′-3′ antisense: CATTGGATTATTTCCCAGGG); *EP300* (5′-3′ sense: TGCAGGCATGGTTCCAGTTT; 5′3′ antisense: AGGTAGAGGGCCATTAGA AGTCA); *EZH2* (5′-3′ sense: GCTGGAATCAAAGGA TACAGACA; 5′-3′ antisense: GACACCGAGAATTTGCTTCAG); *GAPDH* (5′-3′ sense: GTCTCCTCTGACTTCAACAGCG; 5′-3′ antisense: ACCACCCTGTTGCTGTAGCCAA); *KDM6A* (5′-3′ sense: GAGGGAAGCTCTCATTGCTG; 5′-3′ antisense: AGATGAGGCGGATGGTAATG); *KDM6B* (5′-3′sense: CTCAACTTGGGCCTCTTCTC; and 5′-3′antisense: GCCTGTCAGATCCCAGTTCT). For the PCR array experiments, gene expression was assessed using the same cDNA with a Rotor-Gene, RT2 SYBR (Qiagen, Hilden, Germany)^®^ Green qPCR Mastermix (Cat. No. 330529), and a Custom Human RT2 Profiler PCR Array (CLAH47257; Qiagen, Hilden, Germany). This array included 90 genes associated with pro- and anti-adipogenic and pro- and anti-lipogenic pathways, lipolysis, browning, adipokines, receptors, and components of adipocyte signal transduction pathways, as previously reported [18]. A complete list of the genes in the Custom Human RT2 Profiler PCR Array is provided in the Supplementary Materials (Table S1). The CT values were exported and uploaded to the manufacturer’s data analysis web portal at http://www.qiagen.com/geneglobe (accessed on 13 June 2024). The samples were categorized into control and test groups, and the CT values were normalized based on manually selected reference genes. The fold change was also calculated using the 2^(−ΔΔCt)^ method via the data analysis web portal and exported from GeneGlobe^®^ (Qiagen, Hilden, Germany).

### 2.7. Western Blot Analysis

Proteins were extracted according to a previously described protocol [33]. A volume corresponding to 20–30 μg of protein per sample, quantified by a Pierce BCA Protein Assay Kit (Thermo Fisher Scientific) method, was applied for the Western blot analysis. The samples were heated (100 °C for 5min), applied to 10% or 12% polyacrylamide gels, and transferred to a 0.45 µm pore size nitrocellulose membrane (Whatman, Maidstone, UK). The transfer efficiency was determined using a Ponceau solution. After 1 h in a blocking buffer (TBS-T 0.1%) with 5% BSA, the membranes were incubated overnight at 4 °C with the respective primary antibodies (anti-H3K27ac #ab4729, H3K27me3 #sab5700166, anti-ACL #ab40793, anti-KDM6B #ab169197, and anti- p-Nfkb p105/50 #ab28849; Abcam, Waltham, MA, USA; dilution 1:1000), diluted in TBS-T with 2.5% BSA. After three washing steps with TBS-T 0.1%, the membranes were incubated for 1 h at room temperature with a secondary antibody (anti-Rabbit IgG conjugated with horseradish peroxidase), diluted 1:5000 in TBS-T with 2.5% BSA, and then submitted to three additional washing steps with TBS-T. The protein bands were detected by chemiluminescence using an ECL Select Western Blotting Detection Reagent (Cytiva, Amersham, UK), with immediate exposure to the imaging system to capture the images. The ImageJ software (ImageJ 1.54d/Java 1.8.0_345 (64-bit))was used to quantify the bands.

### 2.8. Statistical Analysis

The data analysis was conducted using a one-way Analysis of Variance (ANOVA), followed by a Tukey’s post-test for intergroup comparisons or Student’s *t*-test for comparisons between two groups. The results are presented as the mean ± standard error of the mean (SEM), with significance defined as *p* < 0.05. The statistical evaluations were carried out using GraphPad Prism software, version 9.1.2 (GraphPad Software Inc., San Diego, CA, USA).

## 3. Results

### 3.1. Immunophenotyping of ASCs by Flow Cytometry

**Figure 1:**
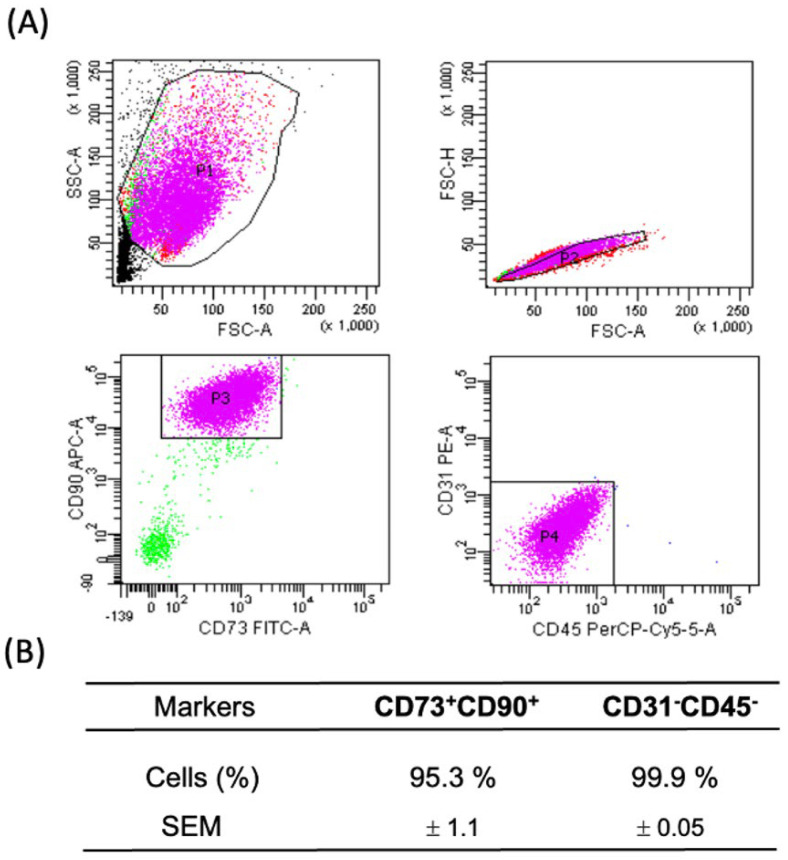
Immunophenotyping of ASCs from human visceral WAT between passages 2 and 4. (**A**) The flow cytometry representation of one experiment, displaying the cell population (P) in the tube with markers P3 (CD73^+^CD90^+^) and P4 (CD31^−^CD45^−^). (**B**) Population values, expressed as percentages. The data represent the mean ± SEM of three independent experiments.

We performed the immunophenotyping of the ASCs using flow cytometry, following culturing in plates that isolate mesenchymal cells from the visceral WAT stromal fraction. This procedure was crucial to confirm the selection of the mesenchymal cells used in the present study. We utilized a panel of specific markers, including CD73-FITC, CD90-APC, CD45-PERCP/CY5, and CD31-PE. CD45 was used to identify the hematopoietic lineage, while CD31 identified endothelial cells. The CD90+CD73+ phenotype was used to identify the mesenchymal cell population. It is important to note that phenotypic markers may vary during cell culture, due to the preferential growth of subpopulations or external factors such as the culture medium used, which can induce changes in marker expression [34,35,36]. Therefore, we performed immunophenotyping between passages P2 and P4, which were the passages used in our studies. The analysis showed satisfactory homogeneity in the mesenchymal markers (positivity for CD90 and CD73 and negativity for CD31 and CD45), even after four passages of the cell culture (Figure 1).

### 3.2. Expression of NF-kB by ASCs Exposed to LPS and/or FO for 72 H

We first validated the effect of LPS in activating the NF-kB pathway in human ASCs, as has been well described in both macrophages and adipocytes [15,37]. The 90% confluence ASCs were treated with LPS, with or without FO, for 72 h. After this period, the secretion medium was collected, and the proteins were extracted for the Western blot analysis.

**Figure 2:**
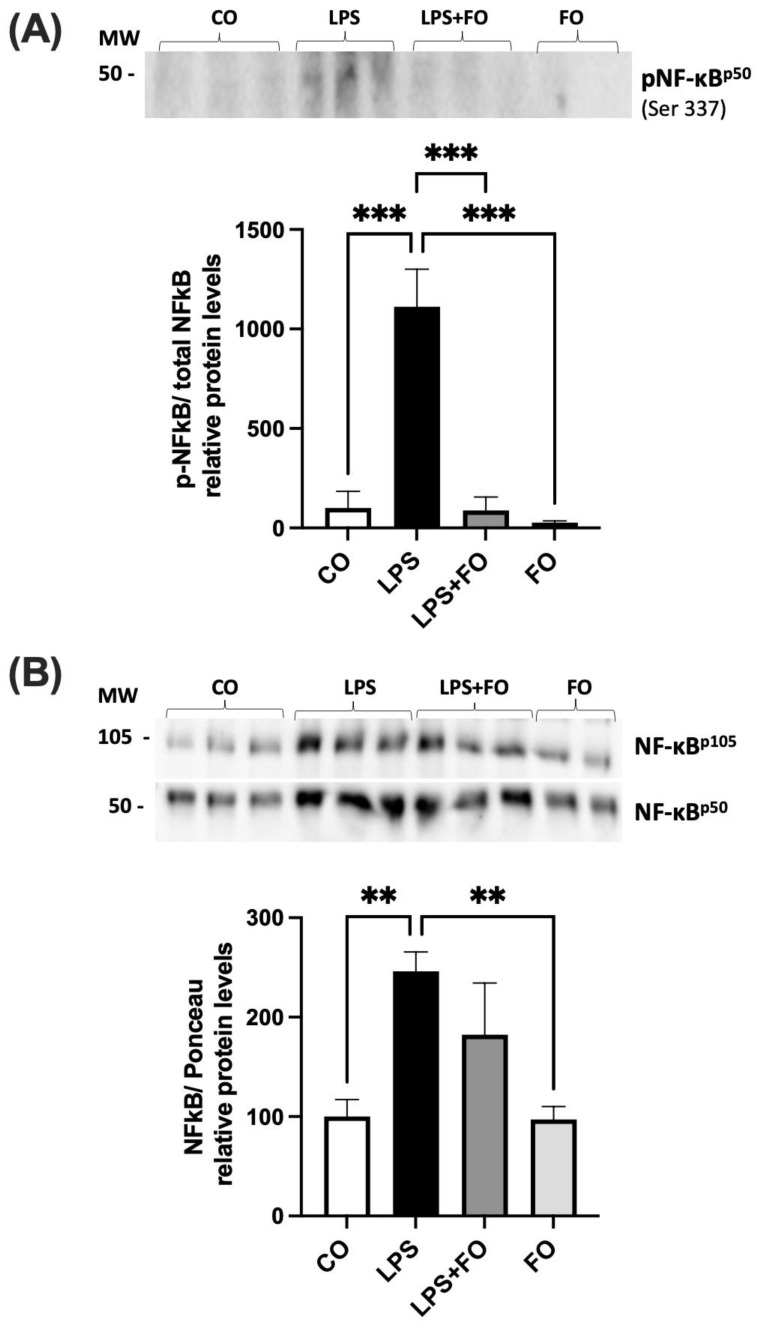
The graphical representation of the phosphorylated NF-kBp50 protein content (**A**) and the total NF-kBp105 and NF-kBp50 (**B**) in ASCs extracted from human visceral WAT, cultured until reaching 90–100% confluence and exposed for 72 h to lipopolysaccharide (LPS, 1 μg/mL), in the presence (or absence) of fish oil (FO, 50 μM). The values are expressed as the mean ± SEM relative to the control and normalized to the total protein, determined by Ponceau staining. All the membranes stained with Ponceau and the corresponding densitometry readings are provided in the Supplementary Materials (Figure S1). Above each graph, a representative autoradiogram of experiments *(n* = 3), quantified by ImageJ, is shown. ** *p* < 0.001, *** *p* < 0.0001. Statistical analyses were conducted using one-way ANOVA.

As a result, we observed an increase in the phosphorylation of NF-kBp50, as well as elevated levels of total NF-kBp105 and NF-kBp50 upon LPS stimulation. Interestingly, these effects were prevented by the presence of FO (Figure 2).

### 3.3. Expression of Histone Modifiers by ASCs Exposed to LPS and/or FO for 72 H

We next evaluated the expression of histone modifiers as potential targets responding to the activation or attenuation of the NF-kB pathway.

**Figure 3:**
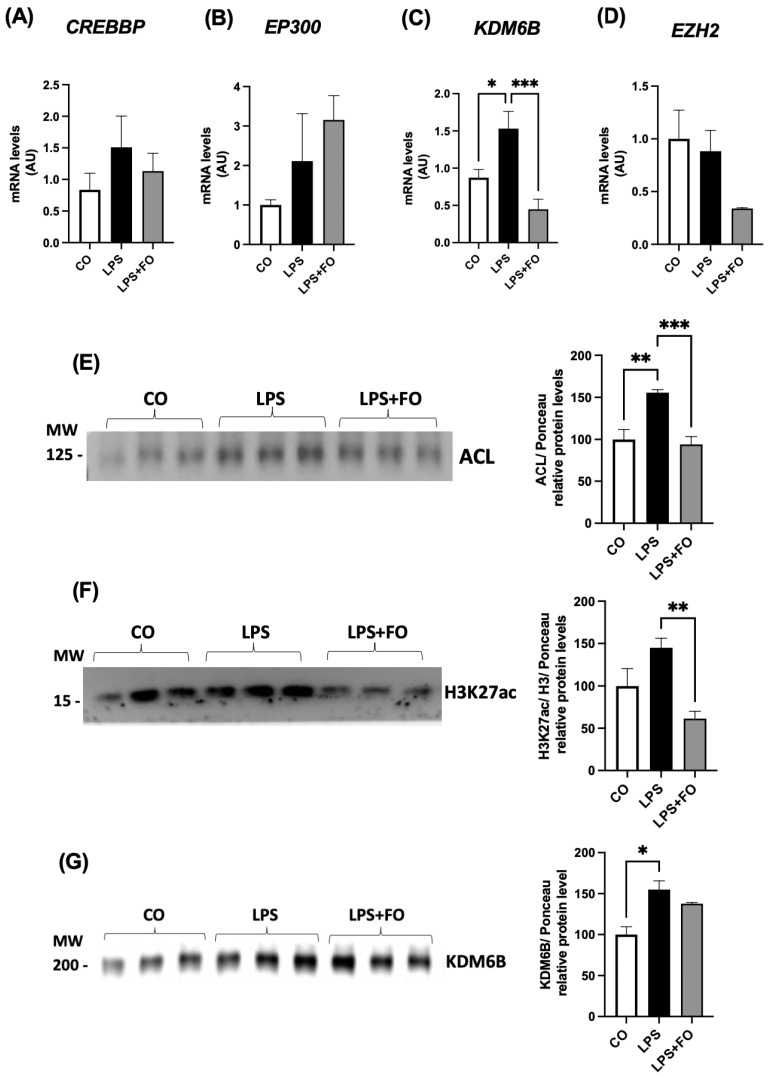
Expression of genes encoding H3K27 modifiers associated with acetylation, *CREBBP* (**A**) and *EP300* (**B**), demethylation, *KDM6B* (**C**), and methylation, *EZH2* (**D**). The graphical representation of the protein content of ACL (**E**), H3K27ac (**F**), and KDM6B (**G**). The ASCs were extracted from human visceral WAT, cultured until reaching 90–100% confluence, and exposed for 72 h to LPS (1 μg/mL), in the presence (or absence) of FO (50 μM). The values are expressed as the mean ± SEM relative to the control and normalized to the expression of constitutive *GAPDH* (**A**–**D**) or the total protein, determined by Ponceau staining (**E**–**G**). All the membranes stained with Ponceau and the corresponding densitometry readings are provided in the Supplementary Materials (Figure S2). Next to each graph, a representative autoradiogram of the experiments (*n* = 3), quantified by ImageJ, is shown. * *p* < 0.05, ** *p* < 0.001, *** *p* < 0.0001. Statistical analyses were conducted using one-way ANOVA.

The gene expression of genes encoding the acetylases CREBBP and EP300 and the methylase EZH2 did not exhibit statistical differences between the groups (Figure 3A,B,D, respectively). The mRNA content for demethylase *KDM6B* was significantly increased in the ASCs treated with LPS and completed inhibited in the LPS+FO group (Figure 3C).

We then investigated the expression of the ATP citrate lyase (ACL) enzyme in the ASCs exposed to LPS with/without FO for 72 h. There was a significant and pronounced increase in *ACL* expression (50%) in response to LPS stimulation, an effect completely prevented in the presence of FO (Figure 3E). ACL catalyzes the transformation of citrate and coenzyme A (CoA) into oxaloacetate and acetyl-CoA. This enzyme is found in both the cytoplasm and the nucleus of various mammalian cells and is the main enzyme involved in the production of acetyl-CoA. It serves not only as a substrate for de novo lipogenesis but also for histone H3 acetylation (via acetylases) in the nucleus of mammalian cells. In the absence of ACL, a significant reduction in histone acetylation occurs, along with the impaired differentiation of cells into adipocytes, reduced expression of Glut4, and lower levels of other glycolytic enzymes. This suggests a correlation between the cell’s energy metabolism and the dynamic regulation of histone acetylation, dependent on the nuclear concentration of acetyl-CoA produced by ACL.

Alongside the rise in ACL, LPS also resulted in an increase in H3K27ac expression (Figure 3F). Once again, FO was able to fully mitigate this effect. Additionally, the exposure of the ASCs to LPS for 72 h significantly increased the expression of the demethylase KDM6B (Figure 3G).

### 3.4. Expression of Genes Encoding Histone Modifiers by ASCs Exposed to LPS and/or NF-κB Inhibitor JSH-23

To confirm that the NF-κB transcription factor alters the expression of H3K27 modifiers and, consequently, the deposition of epigenetic marks on this histone, we conducted experiments in the presence of the NF-κB inhibitor JSH-23, a cell-permeable diamine compound that selectively blocks the nuclear translocation of the p65/p50 NF-κB dimer to the nucleus and its transcriptional activity without affecting IκB-α degradation. The dosage was based on previous studies [31].

**Figure 4:**
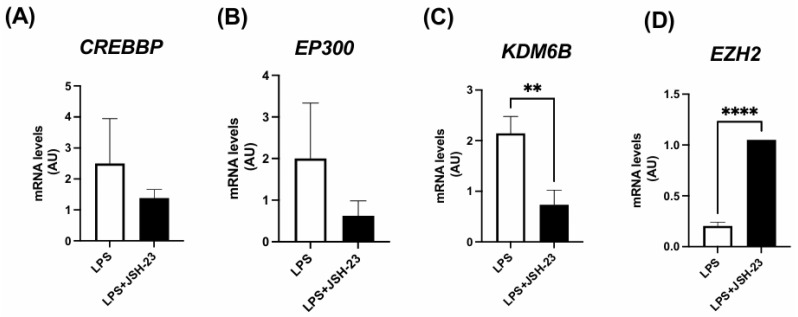
Expression of genes encoding H3K27 modifiers associated with acetylation, *CREBBP* (**A**) and *EP300* (**B**), demethylation, *KDM6B* (**C**), and methylation, *EZH2* (**D**). The ASCs were extracted from human visceral WAT, cultured until reaching 90–100% confluence, and exposed for 72 h to LPS (1 μg/mL), in the presence (or absence) of JSH-23 (10 μM). The values are expressed as the mean ± SEM relative to the control and normalized by the expression of constitutive *GAPDH (n* = 6). ** *p* < 0.001, **** *p* < 0.0001. Statistical analyses were conducted using Student’s *t*-test.

As shown in Figure 2, LPS exposure led to an increase in H3K27 acetyl marks, which was associated with altered expression of H3K27 modifiers. However, the expression of these H3K27 modifiers was significantly modulated in ASCs treated with LPS + JSH-23, compared to cells treated with LPS alone (Figure 4). JSH-23 significantly reduced LPS-induced *KDM6B* mRNA levels (Figure 4C) by inhibiting NF-κB transcriptional activity. Curiously, NF-κB inhibition resulted in the increased expression of *EZH2* on LPS-treated cells (Figure 4D).

### 3.5. Gene Expression by PCR Array

Our next question was whether the effects of the pro-inflammatory agent LPS could be propagated through epigenetic changes in the deposition of the H3K27 mark in ASCs leading to differentiated adipocytes. To investigate this, the ASCs were pre-exposed to LPS for 72 h, then differentiated and analyzed on day 4 post-differentiation. During this period, from day 0 (the induction of differentiation) to day 4, the cells were cultured in the absence of LPS. Adipocyte differentiation was confirmed by light microscopy on day 4 (D4), as evidenced by changes in cell morphology (the cells became rounded) and the formation of intracellular lipid droplets. Additionally, the expression of late-stage differentiation markers, such as leptin, FABP4, Lipe, and FAS (listed in Table 1), which are specific to mature adipocytes, further confirmed the differentiation.

We examined the gene expression profile in these adipocytes. A custom panel containing 90 genes of interest for human adipocytes was designed on a PCR array plate. The differentially expressed genes (both positively and negatively regulated) in response to LPS stimulation are shown in Table 1.

According to the table, the majority of the genes were affected (59 genes), exhibiting a fold change of greater than two, although the *p*-value exceeded 0.5 due to a low number of biological replicates. Nonetheless, all these genes, whose expression was influenced by prior exposure to LPS, play a significant role in the metabolism and secretory function of developing adipocytes. While the validation of the expression of specific genes is crucial following this screening, our results indicate that pre-exposing ASCs to LPS was sufficient to induce the increased expression of genes encoding pro/anti-adipogenic factors, cytokines, receptors, and the components of pro-inflammatory pathways in the newly differentiated adipocytes.

Furthermore, there was a reduction in the expression of LEP and FABP4, key adipocyte markers after differentiation induction, suggesting a delay in the process compared to the control group. There was also a decrease in the expression of pro-browning genes (*SIRT3*, *SRC*, *TBX1*, *TFAM*) and of *PPARGC1B*, which encodes PGC-1β, a protein that plays a crucial role in metabolic regulation and mitochondrial biogenesis. Additionally, we observed a decline in the expression of the components of the insulin signaling pathway (*IRS1*, *IRS2*, *AKT2*).

These results suggest that the chronic exposure of ASCs to LPS prior to differentiation induction may result in an epigenetic memory in these cells, leading to significant changes in the transcription of genes that will only be expressed in adipocytes.

### 3.6. The Expression of Histone Modifiers by the Adipocytes Differentiated from LPS-Pre-Exposed ASCs (In the Presence or Absence of FO) Prior to Differentiation

To further investigate whether the alterations in the expression of several key adipocyte genes is associated with persistent changes in epigenetic marks on H3K27, we examined the expression of the histone modifiers and the acetylation and methylation marks in newly differentiated ASCs.

**Figure 5:**
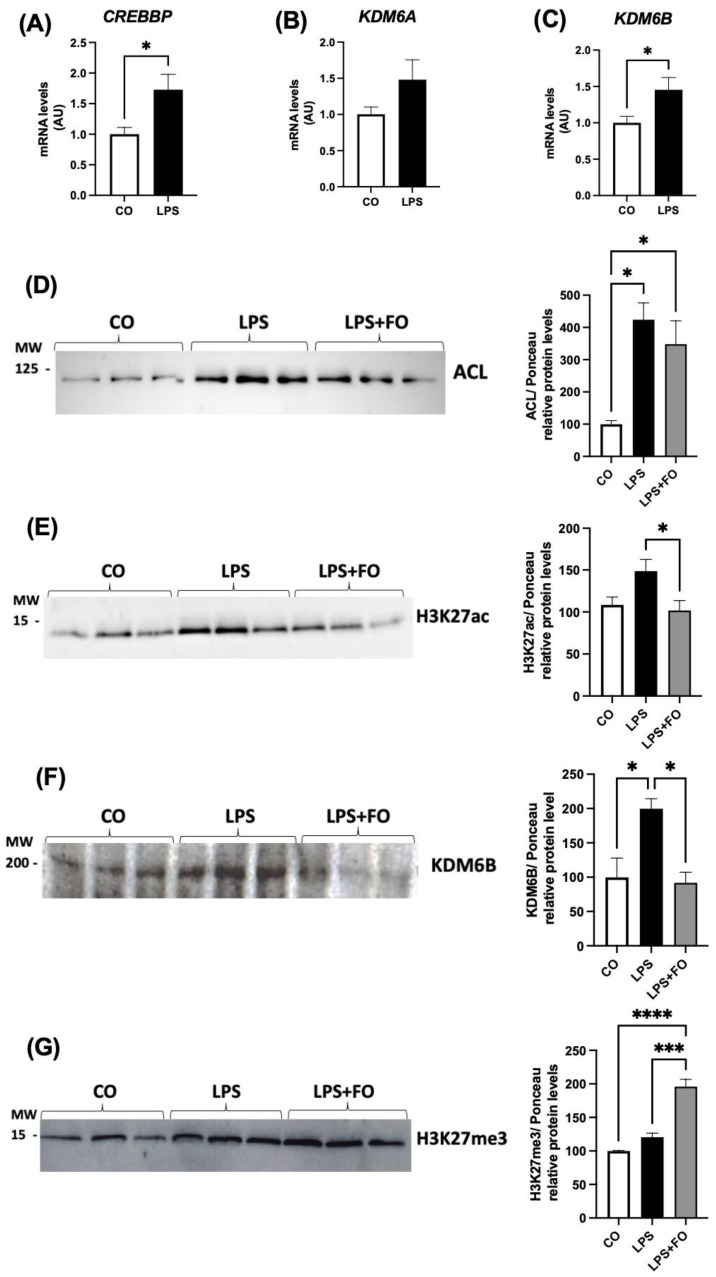
Expression of genes encoding H3K27 modifiers associated with: acetylation, *CREBBP* (**A**), and demethylation, *KDM6A* and *KDM6B* (**B**–**C**), in adipocytes newly differentiated in vitro from visceral ASCs, pre-stimulated chronically with LPS. The graphical representation of the protein content of ACL (**D**), H3K27ac (**E**), KDM6B (**F**), and H3K27me3 (**G**). The cells were cultured until reaching 90–100% confluence and exposed for 72h to LPS (1 μg/mL) in the presence (or absence) of FO (50 μM). After 72h (day 0), the treatment was removed and the cells were washed and differentiated with an adipogenic cocktail until day 4, when the total RNA and protein were extracted and subjected to RT-PCR or Western blotting, respectively. The values are expressed as the mean ± SEM relative to the control and normalized to the expression of constitutive *GAPDH* (**A**–**C**) or the total protein, determined by Ponceau staining (**D**–**G**). All the membranes stained with Ponceau and the corresponding densitometry readings are provided in the Supplementary Materials (Figure S3). Next to each graph, a representative autoradiogram of experiments (*n* = 3), quantified by ImageJ, is shown. * *p* < 0.05, *** *p* < 0.0001, **** *p* < 0,00001. Statistical analyses were conducted using Student’s *t*-test (**A**–**C**) or one-way ANOVA (**D**–**G**).

Our results demonstrated that the effects of LPS on the expression of the genes encoding H3K27 modifiers also persisted after the differentiation, including both of the demethylases *KDM6A* and *KDM6B*, as well as the acetylase *CREBBP* (Figure 5A–C). In agreement with this scenario (increased acetylase and decreased demethylases), we detected an increased deposition of acetylation marks in the newly differentiated ASCs, as evidenced by an upregulation of ACL and H3K27ac (Figure 5D–E, respectively).

Finally, we explored whether FO could attenuate the changes induced by pre-exposing ASCs to LPS prior to differentiation. FO was combined with LPS during the pre-stimulation of ASCs, following the same experimental protocol. Specifically, ASCs were pre-exposed to LPS in the presence or absence of FO for 72 h, followed by differentiation. Cells were analyzed on day 4 post-differentiation, at which point, the treatments were no longer present in the culture. At this stage, we assessed only the lasting effects, focusing on the deposition of epigenetic marks on H3K27. We observed that in the presence of FO, the effects of LPS were attenuated not only in the expression of both ACL and H3K27ac (Figure 5D,E, respectively) but also in demethylase enzyme expression KDM6B (Figure 5F). Thus, FO reduced the LPS-induced acetylation, even after its removal, promoting an increase in H3K27 methylation, that is, increased H3K27me3 (Figure 5G), likely by attenuating the action of the KDM6B demethylase. Therefore, we found that FO’s protective capacity against the effects of LPS persisted under these conditions, protecting adipocyte progenitor cells from the adverse impacts of this pro-inflammatory agent, commonly linked to obesity-related complications.

## 4. Discussion

The main objective of this study was to investigate whether NF-κB transcription factors alter the deposition of methylation and acetylation marks on histone H3K27 in human ASCs and whether FO mitigates these effects. Our results showed that the LPS-induced activation of the NF-κB pathway leads to significant changes in epigenetic marks, including increased H3K27 acetylation, which were effectively attenuated by FO. These findings support the hypothesis that EPA-rich FO acts as an anti-inflammatory agent that is capable of modulating the epigenetic mechanisms involved in inflammation-related dysfunction in WAT.

Several aspects of our findings are consistent with previous studies that have established the role of chronic low-grade inflammation in driving WAT dysfunction, particularly in the context of obesity. Increased plasma LPS, a component of the bacterial cell wall, has been shown to mediate obesity-induced metabolic dysregulation by binding to Toll-like receptor 4 (TLR4). This triggers a cascade of inflammatory responses that significantly affect WAT, promoting insulin resistance, lipid dysregulation, and adipocyte dysfunction. Our study extends these findings by showing that these inflammatory processes also affect the epigenetic marks in the ASC, specifically through changes in H3K27 acetylation and methylation.

We also hypothesized that ASCs challenged with LPS would differentiate into adipocytes with a different transcriptome than the adipocytes derived from unchallenged ASCs. Therefore, we pre-exposed ASCs to LPS, followed by the induction of differentiation into adipocytes, and analyzed the expression of several key adipocyte genes. We also examined the expression of H3K27 histone modifiers and the acetylation and methylation marks in newly differentiated ASCs. Our results showed that the effects of LPS on the expression of genes encoding H3K27 modifiers also persisted after adipogenesis, including the increased expression of KDM6B and H3K27ac.

Therefore, LPS would establish an epigenetic memory in ASCs during the inflammatory process. This memory is evidenced by the altered expression of key adipogenic and metabolic genes, including reductions in *LEP* and *FABP4*—markers of mature adipocytes—and *PPARGC1B*, a regulator of mitochondrial biogenesis and metabolic homeostasis. These changes suggest that chronic inflammation in ASCs affects not only their immediate response to inflammatory stimuli but also affects their long-term differentiation capacity and functionality.

In addition, the upregulation of histone modifiers such as KDM6B, CREBBP, and EZH2 in response to LPS exposure highlights the complex interplay between inflammation and epigenetic regulation. These enzymes are critical in the dynamic addition and removal of epigenetic marks that control gene expression. Although EZH2 is implicated in this process, further investigation, including ChIP-PCR analyses, is needed to clarify its role. In the absence of FO, LPS treatment increased H3K27 acetylation (H3K27ac) and consequently decreased H3K27me3 (triggered by the increased activity of the demethylase KDM6B), which is associated with the transcriptional activation and repression, respectively, of key genes involved in adipocyte metabolism and differentiation [3,34,35].

In this context, changes of histone modification marks mediated by H3K27 modifiers [4] play a critical role at key stages during differentiation. These modifications are essential for the establishment of the adipocyte-selective gene program driven by PPARγ and C/EBPs [38].

The protective effect of FO on LPS-induced epigenetic changes in ASCs highlights its potential role in combating obesity-associated inflammation. This anti-inflammatory property of FO has been demonstrated in various tissues, and our study adds valuable insights into its mechanism of action at the epigenetic level. In particular, the ability of FO to both reduce H3K27ac and restore H3K27me3 in pre-differentiated ASCs suggests that it can re-establish epigenetic balance, which may have far-reaching consequences for adipocyte metabolic health and overall tissue function.

Taken together, our results suggest that the epigenetic modifications observed in LPS-stimulated ASCs are linked to lasting alterations in the expression of genes related to adipogenesis, metabolic regulation, and inflammation, even after differentiation. Remarkably, FO treatment effectively counteracted these LPS-induced changes. These findings highlight the therapeutic potential of FO in reducing inflammation-related epigenetic changes in ASCs and supporting proper adipocyte function.

An important avenue for future research is to explore whether other dietary or pharmacological interventions can similarly modulate the epigenetic profile of ASCs. This may provide new therapeutic strategies to prevent or reverse WAT dysfunction in obesity, metabolic syndrome, and type 2 diabetes. In addition, the exact molecular mechanisms by which FO exerts its protective effects remain to be fully elucidated.

Furthermore, our findings regarding the epigenetic memory established in ASCs following LPS exposure raise critical questions about the long-term effects of inflammation on adipose progenitor cells. Future research should investigate whether this memory can be completely reversed or whether it leads to permanent dysfunction in adipocytes, which may contribute to the chronic nature of obesity-related metabolic diseases. Understanding the reversibility of these epigenetic marks will be critical for developing targeted interventions to restore healthy adipocyte function.

Our study provides compelling evidence that the LPS-induced activation of the NF-κB pathway alters the epigenetic landscape of human ASCs, specifically by modifying the H3K27 acetylation mark. This alteration led to permanent changes in the expression of pivotal genes for TAB function, even after differentiation. FO effectively mitigates these changes, highlighting its potential as a therapeutic agent in the context of obesity-related inflammation. By modulating epigenetic regulators, FO may provide a novel approach to improving adipose tissue function and preventing metabolic dysfunction in individuals with chronic inflammation. These findings contribute to future investigations into the role of epigenetic modifications in WAT biology and the development of novel therapeutic strategies for obesity and related disorders.

## 5. Conclusions

This study provides a significant contribution to understanding the effects of inflammation and FO on the expression of histone modifiers and the epigenetic signatures of transcription factors in human ASCs. These findings offer valuable insights for future research, which may further explore the mechanisms involved and have relevant implications for the development of new therapeutic approaches.

## Figures and Tables

**Table 1:** The list of genes that were up-regulated and down-regulated in the LPS-stimulated group, compared to the control (non-stimulated).

Gene	Ref Seq Number	Fold Regulation	*p*-Value	Pathway Related
Up-regulated				
*GPD1*	NM_005276	3.83	0.372275	Lipases and lipogenic enzymes
*LIPE*	NM_005357	657.62	0.053407	Lipases and lipogenic enzymes
*LPL*	NM_000237	5.08	0.357196	Lipases and lipogenic enzymes
*LPIN1*	NM_145693	2.24	0.035099	Lipases and lipogenic enzymes
*FASN*	NM_004104	2.05	0.198777	Lipases and lipogenic enzymes
*ADRB2*	NM_000024	26.25	0.153417	Anti-adipogenesis
*CDKN1B*	NM_004064	257.98	0.326792	Anti-adipogenesis
*DLK1*	NM_003836	24.27	0.367295	Anti-adipogenesis
*FOXO1*	NM_002015	7.55	0.344553	Anti-adipogenesis
*SIRT1*	NM_012238	6.62	0.283462	Anti-adipogenesis
*WNT3A*	NM_033131	454.39	0.126152	Anti-adipogenesis
*GATA2*	NM_032638	255,172.11	0.135933	Anti-adipogenesis
*CPT1B*	NM_004377	6.17	0.375312	Pro-Browning, fatty acid thermogenesis and oxidation
*DIO2*	NM_000793	639.64	0.136301	Pro-Browning, fatty acid thermogenesis and oxidation
*PRDM16*	NM_199454	5.25	0.074225	Pro-Browning, fatty acid thermogenesis and oxidation
*UCP1*	NM_021833	33.73	0.018196	Pro-Browning, fatty acid thermogenesis and oxidation
*WNT5A*	NM_003392	2.29	0.376944	Pro-Browning, fatty acid thermogenesis and oxidation
*NCOR2*	NM_006312	2.32	0.033524	Anti-Browning
*NCOA2*	NM_006540	11.89	0.225568	Anti-Browning
*NR1H3*	NM_005693	4.93	0.360812	Anti-Browning
*RB1*	NM_000321	3.52	0.045966	Anti-Browning
*ADIPOR2*	NM_024551	75.38	0.141593	Adipokines receptors
*ADRB1*	NM_000684	66,502.05	0.135936	Adipokines receptors
*CXCL10*	NM_001565	7.37	0.174107	Cytokines, growth factors and signal transduction
*IFNG*	NM_000619	6.59	0.179346	Cytokines, growth factors and signal transduction
*IL4*	NM_000589	326.54	0.361392	Cytokines, growth factors and signal transduction
*IL6*	NM_000600	830.95	0.011242	Cytokines, growth factors and signal transduction
*IL10*	NM_000572	25.07	0.361432	Cytokines, growth factors and signal transduction
*IL12B*	NM_002187	985,920.03	0.135932	Cytokines, growth factors and signal transduction
*IL13*	NM_002188	37,086.62	0.103510	Cytokines, growth factors and signal transduction
*TGFB1*	NM_000660	323.16	0.372799	Cytokines, growth factors and signal transduction
*TNF*	NM_000594	11.09	0.050047	Cytokines, growth factors and signal transduction
*INSR*	NM_000208	2.45	0.206700	Cytokines, growth factors and signal transduction
*PTPN1*	NM_002827	2.97	0.262779	Cytokines, growth factors and signal transduction
*IKBKB*	NM_001556	6064.24	0.373517	Cytokines, growth factors and signal transduction
*MAPK8*	NM_002750	4994.45	0.373575	Cytokines, growth factors and signal transduction
*NFKB1*	NM_003998	1,356,174.24	0.035932	Cytokines, growth factors and signal transduction
*ACLY*	NM_001096	2.96	0.281940	Cytokines, growth factors and signal transduction
*CD68*	NM_001251	9.49	0.368878	Cytokines, growth factors and signal transduction
Down-regulated				
*LEP*	NM_000230	−2.18	0.0858765	Adipokines
*FABP4*	NM_001442	−3.72	0.0652578	Pro-adipogenesis
*KLF4*	NM_004235	−2.55	0.239165	Pro-adipogenesis
*WNT1*	NM_005430	−10.84	0.460085	Anti-adipogenesis
*KLF2*	NM_016270	−19.14	0.241078	Anti-adipogenesis
*NRF1*	NM_005011	−3.33	0.295323	Pro-Browning, fatty acid thermogenesis and oxidation
*PPARGC1B*	NM_133263	−7.18	0.012021	Pro-Browning, fatty acid thermogenesis and oxidation
*SIRT3*	NM_012239	−4.66	0.361493	Pro-Browning, fatty acid thermogenesis and oxidation
*SRC*	NM_005417	−102.81	0.372853	Pro-Browning, fatty acid thermogenesis and oxidation
*TBX1*	NM_005992	−7.42	0.323127	Pro-Browning, fatty acid thermogenesis and oxidation
*TFAM*	NM_003201	−2.56	0.658977	Pro-Browning, fatty acid thermogenesis and oxidation
*IRS1*	NM_005544	−2.43	0.549537	Cytokines, growth factors and signal transduction
*IRS2*	NM_003749	−63.29	0.000514	Cytokines, growth factors and signal transduction
*AKT2*	NM_001626	−8.47	0.38668	Cytokines, growth factors and signal transduction

Gene expression profile in adipocytes newly differentiated in vitro from human visceral ASCs, pre-stimulated chronically with LPS. The cells were cultured until reaching 90–100% confluence and exposed for 72 h to LPS (1 μg/mL). After 72 h (day 0), the treatment was removed and the cells were washed and differentiated with an adipogenic cocktail until day 4, when the RNA was extracted and subjected to an RT2 Profiler PCR Array. The nomenclature of the genes in the table is described in the Supplementary Materials.

## Data Availability

The data are available from the corresponding author upon specific request.

## Supplementary Materials

The following supporting information can be downloaded at https://www.mdpi.com/article/10.3390/life14121653/s1. Table S1. List of selected genes in Custom Human RT2 Profiler PCR Array. Figure S1. Phosphorylated NF-kBp50^Ser 337^ (A), and total NF-kBp105 and NF-kBp50 (B). Figure S2. ACL (E), H3K27ac (F), and KDM6B (G). Figure S3. ACL (A), H3K27ac (B), KDM6B (C), and H3K27me3 (D).

## Abbreviations

(ACL) ATP citrate lyase; (ASCs) adipose-derived stem cells; (BMI) Body mass index; (BSA) Bovine serum albumine; (cDNA) complementary DNA; (CD90-APC) APC Mouse Anti-Human CD90; (CD45-PERCP/CY5) PerCP-Cy™5.5 Mouse Anti-Human CD45; (CD73-FITC) FITC Mouse Anti-Human CD73; (CD31-PE) PE Rat Anti-Mouse CD31; (CEBPα) CCAAT/enhancer binding protein alpha; (CEP) Research Ethics Committee; (ChIP-seq) Chromatin Immunoprecipitation Sequencing; (CoA) coenzyme A; (CREBBP or CBP) CREB-binding protein; (DHA) docosahexaenoic acid; (D’MEM) Dulbecco’s modified Eagle’s medium; (DNA) Deoxyribonucleic acid; (D4) Day 4 after differentiation; (EHB buffer) EARLE/HEPES salt; (EPA) eicosapentaenoic acid; (EP300 or p300) E1A binding protein p300; (Ezh2) Enhancer of zeste homolog 2; (FABP4) Fatty acid binding protein 4; (FBS) fetal bovine serum; (FO) fish oil; (Glut4 ou SLC2A4) Solute carrier family 2 member 4; (GAPDH) Glyceraldehyde-3-phosphate dehydrogenase; (HFD) high-fat diet; (hMSCs) human mesenchymal stem cells; (H3) histone H3; (H3K27) histone H3 lysine 27; (H3K27ac) Acetylation of histone H3 lysine 27; (H3K27me3) trimethylation of histone H3 lysine 27; (IBMX) 3-isobutyl-1-methylxanthine; (ICF) Informed Consent Form; (IκB-α) NFKB inhibitor α; (IRS1) Insulin receptor substrate 1; (IRS2) Insulin receptor substrate 2; (JSH-23) 4-Methyl-N1-(3-phenylpropyl) benzene-1,2-diamine; (JMJD3) Jumonji domain-containing protein-3, also known as KDM6B; (KDM6A) Lysine-specific demethylase 6A; (KDM6B) Lysine demethylase 6B; (LEP) leptin; (LPS) lipopolysaccharide; (NF-Κb) Nuclear factor kappa B; (n-3 PUFA) long-chain polyunsaturated fatty acids n-3; (PBS) Phosphate Buffered Saline; (PCR) Array Polymerase chain reaction; (PPARGC1B) Peroxisome proliferative activated receptor, gamma, coactivator 1 beta; (PPARγ) Peroxisome proliferator activated receptor gamma; (P1) passage 1; (P2) Passage 2; (P4) Passage 4; (P5) Passage 5; (RNA) Ribonucleic acid; (RT-PCR) Real Time Polymerase chain reaction; (SCR) Rous sarcoma oncogene; (SIRT3) Sirtuin 3 (silent mating type information regulation 2, homolog) 3 (S. cerevisiae); (SVF) stromal cell fraction; (TBS-T) Tris-buffered saline with Tween^®^ 20; (TBX1) T-box 1; (TFAM) transcription factor A; (TLR4) Toll-like receptor 4; (UNIFESP) Federal University of São Paulo; (UTX) Ubiquitously transcribed tetratricopeptide repeat, X chromosome also known as KDM6A; (WAT) white adipose tissue. The nomenclature of the genes in the Table S1 is described in the Supplementary Materials.
