# Supplementary material for: The Activation of the NF-κB Pathway in Human Adipose-Derived Stem Cells Alters the Deposition of Epigenetic Marks on H3K27 and Is Modulated by Fish Oil
Source: Life (Basel). 2024 Dec 12;14(12):1653. doi: 10.3390/life14121653 (PMC11678231; doi:10.3390/life14121653)
Supplement: Supplementary file 1 [file life-14-01653-s001.zip › life-3300984-supplementary.pdf]

SUPPLEMENTARY DATA

Table S1 – List of selected genes in Custom Human RT2 Profiler PCR Array

Pathways	Genes
**Adipokines**	*ADIPOQ, CFD, LEP, RETN*
**Lipases and lipogenic enzymes**	*ACACA, GPD1, LIPE (HSL), SCD, LPL, PNPLA2, LPIN1, PCK1, FASN*
**Pro- adipogenesis**	*CEBPA, CEBPB, CEBPD, PPARG, SREBF1, FABP4, PLIN1, FGF2, FGF10, JUN, LMNA, SFRP1, SLC2A4 (GLUT4), KLF15, KLF4*
**Anti- adipogenesis**	*ADRB2, CDKN1A (P21CIP1, CAP20), CDKN1B (P27KIP1), DDIT3 (GADD153, CHOP), DLK1(PREF1), FOXO1, NCOR2, SHH, SIRT1, WNT1, WNT3A, GATA2, KLF2*
**Pro-Browning, fatty acid thermogenesis and oxidation**	*BMP7, CIDEA, CPT1B, CREB1, DIO2, ELOVL3, FOXC2, MAPK14 (P38ALPHA), NRF1, PPARA, PPARD, PPARGC1A (PGC1ALPHA), PPARGC1B (PERC, PGC1BETA), PRDM16, SIRT3, SRC, TBX1, TFAM, UCP1, WNT5A*
**Anti-Browning**	*NCOA2, NR1H3, RB1, WNT10B*
**Adipokines receptors**	*LEPR, ADIPOR2, ADRB1*
**Cytokines, growth factors and signal transduction**	*CCL2 (MCP1), CXCL10, IFNG, IL1B, IL4, IL6, IL10, IL12B, IL13, TGFB1, TNF, INSR, IRS1, IRS2, AKT2, PTPN1 (PTP1B), IKBKB (IKKbeta), MAPK8 (JNK1), NFKB1, PIK3R1 (P85ALPHA), IRF4, CD68*

***ACACA (ACC1) -** Acetyl-Coenzyme A carboxylase alpha; **ADIPOQ**, Adiponectin; **ADIPOR2** - Adiponectin receptor 2; **ADRB1** - Adrenergic receptor, beta 1; **ADRB2**- Adrenergic receptor, beta 2; **AKT2** - Thymoma viral proto-oncogene 2; **BMP7** - Bone morphogenetic protein 7; **CCL2** - Chemokine (C-C motif) ligand 2; **CD68** - CD68 antigen; **CDKN1A** - Cyclin-dependent kinase inhibitor 1A (P21); **CDKN1B** - Cyclin-dependent kinase inhibitor 1B; **CEBPA or C/EBPA** - CCAAT/enhancer binding protein (C/EBP), alpha; **CEBPB or C/EBPB** - CCAAT/enhancer binding protein (C/EBP), beta; **CEBPD** - CCAAT/enhancer binding protein (C/EBP), delta; **CFD** - Complement factor D (adipsin); <u>CIDEA - Cell death-inducing DNA fragmentation factor, alpha subunit-like effector A;</u> **CPT1B** - Carnitine palmitoyltransferase 1b, muscle; **CXCL10** - Chemokine (C-X-C motif) ligand 10; **DDIT3** - DNA-damage inducible transcript 3; **DIO2** - Deiodinase, iodothyronine, type II; **DLK1** (PREF-1) - Delta-like 1 homolog (Drosophila); **ELOVL3** - Elongation of very long chain fatty acids (FEN1/Elo2, SUR4/Elo3, yeast)-like 3; **FABP4** (aP2) - Fatty acid binding protein 4, adipocyte; **FASN** - Fatty acid synthase; **FGF10** - Fibroblast growth factor 10; **FGF2** - Fibroblast growth factor 2; **FOXC2** - Forkhead box C2; **FOXO1** - Forkhead box O1; **GATA2** - GATA binding protein 2; **GPD1** - Glycerol-3-phosphate dehydrogenase 1 (soluble); **IFNG** - Interferon gamma; **IKBKB(IKKBETA)** - Inhibitor of kappaB kinase beta; **IL10** - Interleukin 10; **IL12B** - Interleukin 12b; **IL13** - Interleukin 13; **IL1B** - Interleukin 1 beta; **IL4** - Interleukin 4; **IL6** - Interleukin 6; **INSR** - Insulin receptor; **IRF4** - Interferon regulatory factor 4; **IRS1** - Insulin receptor substrate 1; **IRS2** - Insulin receptor substrate 2; **JUN** - Jun oncogene; **KLF15** - Kruppel-like factor 15; **KLF2** - Kruppel-like factor 2 (lung); **KLF4** - Kruppel-like factor 4 (gut); **LEP** – Leptin; **LEPR** - Leptin receptor; **LIPE (HSL)** - Lipase, hormone sensitive; **LMNA** - Lamin A; **LPIN1** - Lipin 1; **LPL** - Lipoprotein lipase; **MAPK14** - Mitogen-activated protein kinase 14; **MAPK8(JNK1)** - Mitogen-activated protein kinase 8; **NCOA2** - Nuclear receptor coactivator 2; **NCOR2** - Nuclear receptor co-repressor 2; **NFKB1** - Nuclear factor of kappa light polypeptide gene enhancer in B-cells 1, p105; **NR1H3** - Nuclear receptor subfamily 1, group H, member 3; **NRF1** - Nuclear respiratory factor 1; **PCK1** -Phosphoenolpyruvate carboxykinase 1, cytosolic; **PIK3R1** - Phosphatidylinositol 3-kinase, regulatory subunit, polypeptide 1 (p85 alpha); **PLIN1** - Perilipin 1; **PNPLA2-** patatin like phospholipase domain containing 2; **PPARA** - Peroxisome proliferator activated receptor alpha; **PPARD** - Peroxisome proliferator activator receptor delta; **PPARG or PPARΓ** - Peroxisome proliferator activated receptor gamma; **PPARGC1A** - Peroxisome proliferative activated receptor, gamma, coactivator 1 alpha; **PPARGC1B** - Peroxisome proliferative activated receptor, gamma, coactivator 1 beta; **PRDM16** - PR domain containing 16; **PTPN1** - Protein tyrosine phosphatase, non-receptor type 1; **RB1** - Retinoblastoma 1; **RETN** – Resistin; **SCD** - Stearoyl-Coenzyme A desaturase 1; **SCR** - Rous sarcoma oncogene; **SFRP1** - Secreted frizzled-related protein 1; **SHH** - Sonic hedgehog; **SIRT1** - Sirtuin 1 (silent mating type information regulation 2, homolog) 1 (S. cerevisiae); **SIRT3** - Sirtuin 3 (silent mating type information regulation 2, homolog) 3 (S. cerevisiae); **SLC2A4** (GLUT4) - Solute carrier family 2 (facilitated glucose transporter), member 4;*

SREBF1 - Sterol regulatory element binding transcription factor 1; TBX1 - T-box 1; TFAM - Transcription factor A, mitochondrial; TGFB1 - Transforming growth factor, beta 1; TNF - Tumor necrosis factor; UCP1 - Uncoupling protein 1 (mitochondrial, proton carrier); WNT1 - Wingless-related MMTV integration site 1; WNT10B - Wingless related MMTV integration site 10b; WNT3A - Wingless-related MMTV integration site 3A; WNT5A - Wingless-related MMTV integration site 5A.

Ponceau staining, densitometry readings and molecular weight markers for the protein content

For quantitative protein expression analysis, we normalized total protein using Ponceau S staining, which offers significant advantages over traditional housekeeping proteins. This method provides a rapid, consistent, and protein-load-independent means of visualizing total protein, reducing variations due to experimental conditions and enhancing the accuracy and reliability of quantification. As highlighted by Motiz (2017) [1] (2017, doi:10.1002/pmic.201600189), Ponceau S staining has become a widely adopted normalization technique in recent studies.

The molecular weight marker used in the Western blot experiments was Thermo Scientific Spectra Multicolor Broad Range Protein Ladder (Product #26634), a 4-color (blue, orange, green, pink) marker.

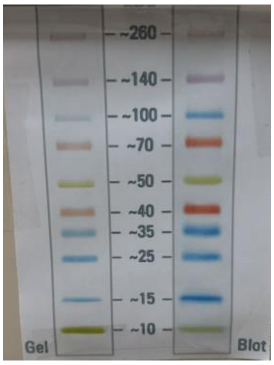

Note: Due to the low molecular weight of histone 3 (15 kDa), we reused the same membrane for the visualization of ACL protein (125 kDa).

Figure S1. Phosphorylated NF-kBp50$^{Ser\ 337}$ (**A**), and total NF-kBp105 and NF-kBp50 (**B**)

A) Phosphorylated Nf-kB p50 $^{Ser\ 337}$

	p-Nfkb p50kDa	CO	CO	CO	LPS	LPS	LPS	LPS+FO	LPS+FO	LPS+FO	FO	FO
	Sample	92,1	114,7	250,3	1076,9	1418,7		170,2	123,1	17,5	21,9	6,1
	PONCEAU	86.366	79.855	52.393	45.371	46.953	45.183	45.320	56.852	38.260	27.706	12.596
densitometry readings	Average											
	RATIO	0,001066	0,001436	0,004777	0,023735	0,030215	0,000000	0,003756	0,002165	0,000457	0,000790	0,000484
	Average of CO	0,002427										
	PERCENTAGE	43,94	59,19	196,87	978,09	1245,12	0,00	154,76	89,23	18,85	32,57	19,96

	CO	LPS	LPS+FO	FO
	43,9441101	978,0946312	154,7581410	32,5727281
	59,1895657	1245,1196072	89,2269720	19,9563463
	196,8663242		18,8485060	
Average	100	1111,607119	87,61120633	32,57272813

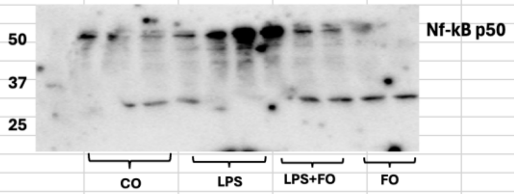

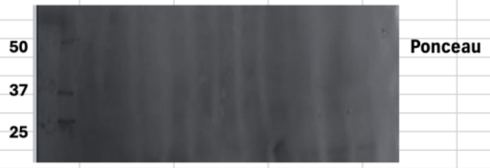

B) Total Nf-kB p105 e NF-kBp50

p-Nfkb p1050kDa	CO	CO	CO	LPS	LPS	LPS	LPS+FO	LPS+FO	LPS+FO	FO	FO
p105 Sample	1.247.355	1.598.770	2.974.891	6.840.669	6.204.912	4.860.669	5.228.184	3.155.477	4.221.841	5.424.719	3.186.062
PONCEAU	86.366	79.855	52.393	75.371	76.953	75.183	75.320	56.852	78.260	77.706	72.596
Average	14,44	20,02	56,78	90,76	80,63	64,65	69,41	55,50	53,95	69,81	43,89
Average of CO	30,41										
PERCENTAGE	47,49	65,83	186,69	298,41	265,11	212,57	228,22	182,49	177,37	229,53	144,30

	CO	LPS	LPS+FO	FO
	47,49	298,41	228	229,53
	65,83	265,11	182	144,30
	186,69	212,57	177	
Average	100,00	258,70	196,03	186,91

p-Nfkb p50kDa	CO	CO	CO	LPS	LPS	LPS	LPS+FO	LPS+FO	LPS+FO	FO	FO
p50 Sample	4.067.912	3.636.376	3.439.841	6.343.205	7.406.740	6.295.912	6.177.154	4.198.205	5.138.598	3.929.719	3.150.376
PONCEAU	86.366	79.855	52.393	75.371	76.953	75.183	75.320	56.852	78.260	77.706	72.596
Average	47,10084987	45,53723624	65,65459126	84,1597564	96,25017868	83,74116489	82,01213489	73,8444558	65,6605929	50,57162896	43,3959998
Average of CO	52,76422579										
PERCENTAGE	89,26663694	86,30323966	124,4301234	159,501547	182,4155993	158,7082225	155,4313243	139,951747	124,441498	95,84453899	82,245118

	CO	LPS	LPS+FO	FO
	89,27	159,50	155,43	95,84
	86,30	182,42	139,95	82,25
	124,43	158,71	124,44	
Average	100,00	166,88	139,94	89,04

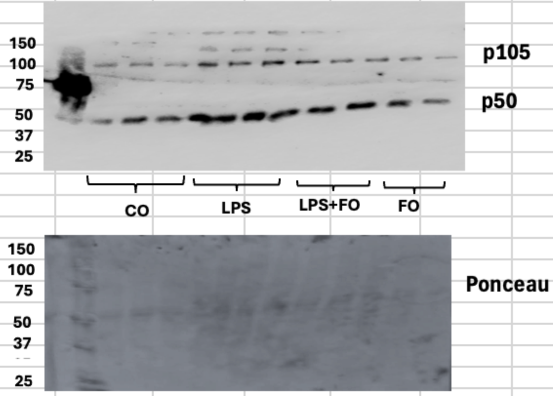

Figure S2. ACL (**A**), H3K27ac (**B**), and KDM6B (**C**)

A) ACL

		94	95	96	98	99	100	102	103	104
	ACL	CO	CO	CO	LPS	LPS	LPS	LPS+FO	LPS+FO	LPS+FO
	Sample	1.270.184	2.227.426	3.262.669	4.648.619	4.319.669	4.680.548	2.897.184	2.495.062	2.117.184
densitometry readings	PONCEAU	56.741.546	70.309.919	79.841.605	91.145.333	93.919.090	92.532.697	86.671.919	84.188.990	80.881.404
	RATIO	0,022385	0,031680	0,040864	0,051002	0,045994	0,050583	0,033427	0,029636	0,026176
	Average of CO	0,03								
	PERCENTAGE	70,74	100,12	129,14	161,18	145,35	159,85	105,64	93,66	82,72

	CO	LPS	LPS+FO
	70,74	161,18	105,64
	100,12	145,35	93,66
	129,14	159,85	82,72
Average	100	155,5	94,0

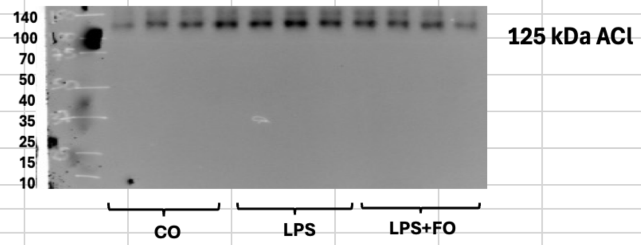

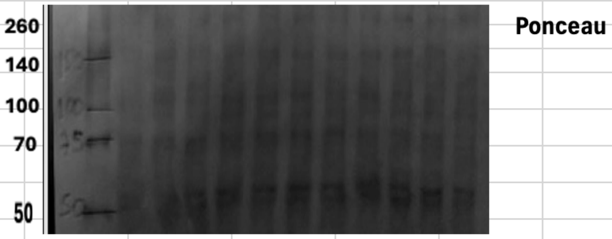

B) H3K27ac

		94	95	96	98	99	100	102	103	104
	H3K27ac	CO	CO	CO	LPS	LPS	LPS	LPS+FO	LPS+FO	LPS+FO
	Sample	4.084.589	12.764.146	10.073.803	10.538.095	12.918.459	15.541.116	7.395.560	5.521.146	3.618.489
densitometry readings	PONCEAU	825.527	2.894.205	1.412.719	1.662.635	1.519.123	1.710.817	2081568,58	1.268.172	1.674.870
	RATIO	4,947856	4,410243	7,130790	6,338190	8,503893	9,084032	3,552878	4,353626	2,160459
	Average of CO	5,50								
	PERCENTAGE	90,02	80,24	129,74	115,32	154,72	165,28	64,64	79,21	39,31

	CO	LPS	LPS+FO
	90,02	115,32	64,64
	80,24	154,72	79,21
	129,74	165,28	39,31
Average	100	145,1	61,1

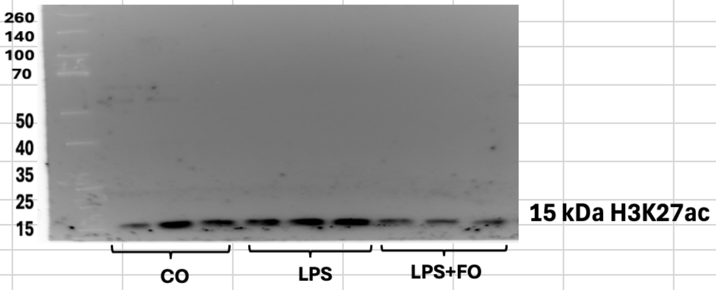

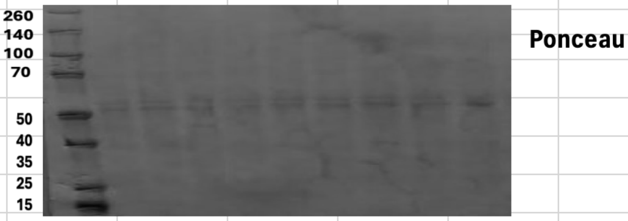

C) KDM6B

	KDM6B	94 CO	95 CO	96 CO	98 LPS	99 LPS	100 LPS	102 LPS+FO	103 LPS+FO	104 LPS+FO
	Sample	6.752.761	9.317.711	10.947.782	11.831.489	13.577.853	16.460.752	17.043.803	12.520.439	12.285.853
densitometry readings	PONCEAU	825527	2894205	1412719	1144080,83	1519123	1710817	2081568,58	1268172	1674870,29
	RATIO	8,1799	3,2194	7,7494	10,3415	8,9380	9,6216	8,1880	9,8728	7,3354
	Average of CO	6,38								
	PERCENTAGE	128,15	50,44	121,41	162,02	140,03	150,74	128,28	154,68	114,92

	CO	LPS	LPS+FO
	128,15	162,02	128,28
	50,44	140,03	154,68
	121,41	150,74	114,92
Average	100	150,9	132,6

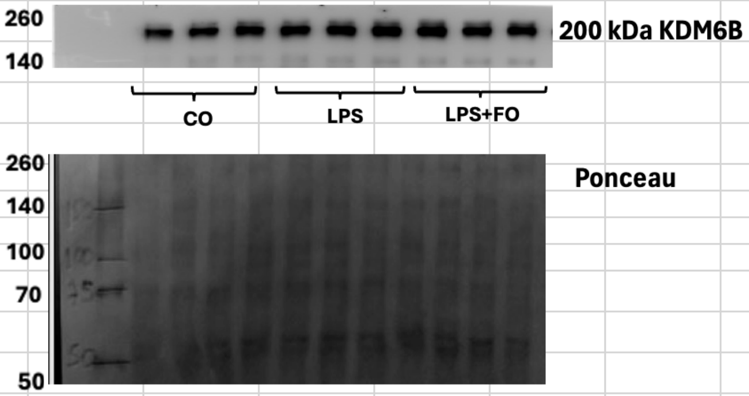

Figure S3. ACL (**A**), H3K27ac (**B**), KDM6B (**C**), and H3K27me3 (**D**)

A) ACL

	ACL	94 CO	95 CO	96 CO	98 LPS	99 LPS	100 LPS	102 LPS+FO	103 LPS+FO	104 LPS+FO
	Sample	1.532.134	2.294.205	2.005.033	6.295.296	9.580.903	7.710.024	6.587.489	5.426.125	3.037.518
densitometry readings	PONCEAU	35000	35610	34056	35008	34608	30633	25880	25880	25880
	RATIO	44	64	59	180	277	252	255	210	117
	Average of CO	55,69								
	PERCENTAGE	78,60	115,68	105,71	322,89	497,09	451,93	457,05	376,47	210,75

	CO	LPS	LPS+FO
	78,60	322,89	457,05
	115,68	497,09	376,47
	105,71	451,93	210,75
Average	100	424,0	348,1

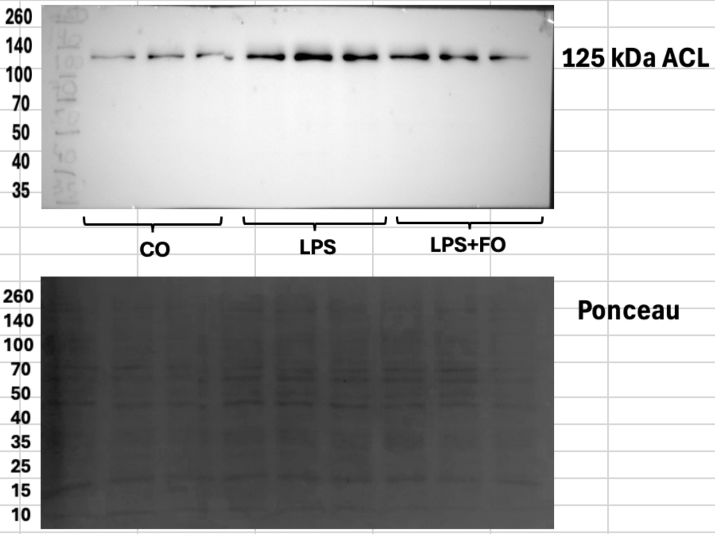

B) H3K27ac

	H3K27ac	CO	CO	CO	LPS	LPS	LPS	LPSOP	LPSOP	LPSOP
	**Sample**	3.128.598	5.057.841	3.536.113	4.001.770	4.265.841	4.248.083	2.109.184	1.739.598	2.116.012
**densitometry readings**	**PONCEAU**	101.851.304	68.903.567	64.692.454	66.161.446	65.001.659	57.759.274	49.483.597	36.664.496	45.723.923
	RATIO	0,030717	0,073405	0,054660	0,060485	0,065627	0,073548	0,042624	0,047446	0,046278
	Average of CO	0,05								
	PERCENTAGE	58,04	138,69	103,27	114,28	123,99	138,96	80,53	89,64	87,44

	CO	LPS	LPSOP
	58,04	114,28	80,53
	138,69	123,99	89,64
	103,27	138,96	87,44
Average	100	125,74	85,87

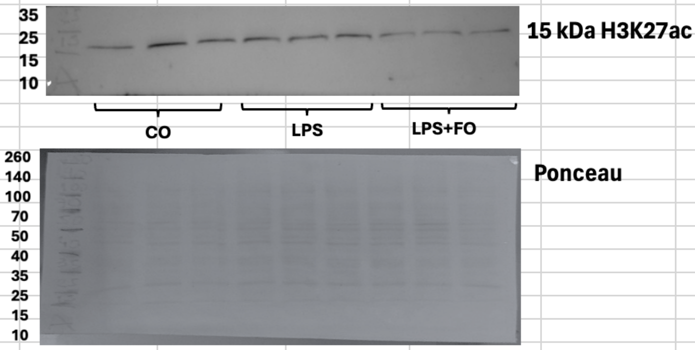

		118	119	120	122	123	124	126	127	128
	**H3K27ac**	CO	CO	CO	LPS	LPS	LPS	LPS+FO	LPS+FO	LPS+FO
	**Sample**	2.357.083	4.033.255	3.202.548	7.176.276	8.042.175	5.354.104	4.981.397	4.289.054	2.151.740
**densitometry readings**	**PONCEAU**	107.171.224	141.100.487	153.262.224	160.712.659	170.434.044	174.682.115	151.414.973	129841685	118264776
	RATIO	0,02199	0,02858	0,02090	0,04465	0,04719	0,03065	0,03290	0,03303	0,01819
	Average of CO	0,023824589								
	PERCENTAGE	92	120	88	187	198	129	138	139	76

CO	LPS	LPS+FO
92,31	187,42	138,09
119,98	198,06	138,65
87,71	128,65	76,37
100	171,38	117,70

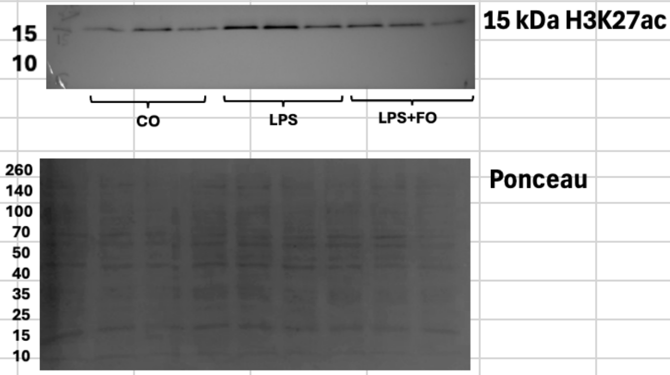

C) KDM6B

		94	95	96	98	99	100	102	103	104
	**KDM6B**	CO	CO	CO	LPS	LPS	LPS	LPS+FO	LPS+FO	LPS+FO
	**Sample**	1.021.355	2.007.598	3.565.740	4.279.740	4.134.962	3.965.740	1.749.669	1.383.134	1.224.690
densitometry readings	**PONCEAU**	80.485.156	101.380.462	104.251.725	111.267.181	91.358.675	79.851.482	65.017.663	72.153.825	80.560.654
	RATIO	0,01269	0,01980	0,03420	0,03846	0,04526	0,04966	0,02691	0,01917	0,01520
	Average of CO	0,02223192								
	PERCENTAGE	57,08	89,07	153,85	173,01	203,58	223,39	121,05	86,22	68,38

	CO	LPS	LPS+FO
	57,08	173,01	121,05
	89,07	203,58	86,22
	153,85	223,39	68,38
Average	100	200,0	91,9

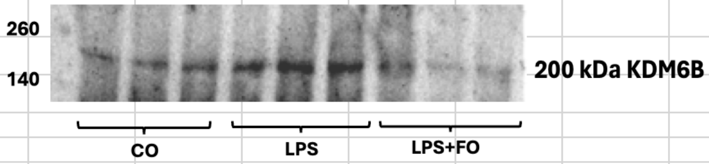

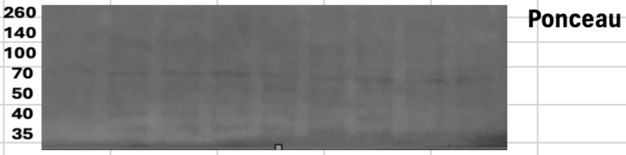

D) H3K27me3

	**H3K27me3**	94 CO	95 CO	96 CO	98 LPS	99 LPS	100 LPS	102 LPSOP	103 LPSOP	104 LPSOP
	**PONCEAU**	2.056.113	2.708.234	1.519.284	2.805.305	3.657.012	4.920.790	5.797.154	3.969.648	4.707.619
densitometry readings	**Sample**	836.113	1.134.577	631.870	1.260.477	2.317.134	2.527.648	4.830.548	3.602.355	3.257.598
	Average of CO	0,40665	0,41894	0,41590	0,44932	0,63361	0,51367	0,83326	0,90747	0,69198
		0,41								
	PERCENTAGE	98,26	101,23	100,50	108,58	153,11	124,13	201,35	219,29	167,22

	CO	LPS	LPSOP
	98,26	108,58	201,35
	101,23	153,11	219,29
	100,50	124,13	167,22
Average	100	128,60	195,95

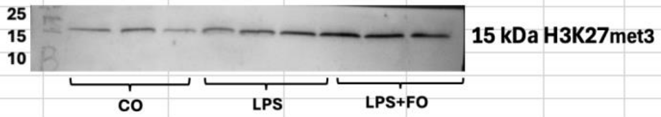

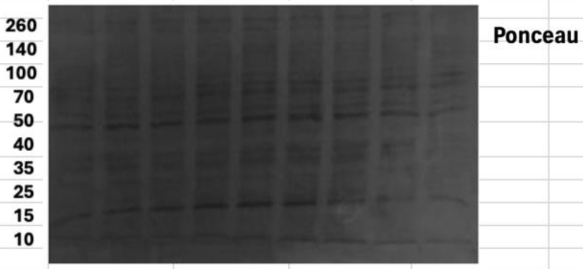

References

1. Moritz CP. Tubulin or Not Tubulin: Heading Toward Total Protein Staining as Loading Control in Western Blots. Proteomics. **2017**, 17, 1600189. https://doi.org/10.1002/pmic.201600189
